# A descriptive study of the single-nucleotide polymorphisms known to affect the Tacrolimus trough concentration per dose, among a population of kidney failure patients in a tertiary hospital in Ghana

**DOI:** 10.1186/s13104-024-06868-8

**Published:** 2024-07-29

**Authors:** Edward Kwakyi, Edmund Tetteh Nartey, Michael Kobina Otabil, Isaac Asiedu-Gyekye, Samuel Yao Ahorhorlu, Vincent Bioma, William Kudzi

**Affiliations:** 1 Department of Medicine, University of Ghana Medical School, Legon, Ghana; 2 Center for Tropical Clinical Pharmacology and Therapeutics, University of Ghana Medical School, University of Ghana, P.O. Box GP 4236, Legon, Accra, Ghana; 3 Department of Pharmacology and Toxicology, School of Pharmacy, University of Ghana, Legon, Ghana; 4 Department of Medicine, University of Ghana Medical School, Legon, Ghana

**Keywords:** Tacrolimus, Single-nucleotide polymorphisms, Korle-bu Teaching Hospital, CYP3A4, CYP3A5, MDR1

## Abstract

**Background:**

The burden of chronic kidney disease (CKD) and kidney failure in Ghana is on the ascendency, with the prevalence of CKD estimated at 13.3%. Patients with CKD who progress to kidney failure require life sustaining kidney replacement therapy (KRT) which is almost exclusively available in Ghana as haemodialysis. Kidney transplantation is considered the best KRT option for patients with irreversible kidney failure due to its relative cost efficiency as well as its superiority in terms of survival and quality of life. However, because transplants may trigger an immune response with potential organ rejection, immunosuppressants such as tacrolimus dosing are required.

**Objective:**

This study sought to determine single nucleotide polymorphisms in CYP3A5, CYP3A4 and MDR1 genes that affect the pharmacokinetics of Tacrolimus in a population of Ghanaian patients with kidney failure.

**Method:**

This cross-sectional study comprised of 82 kidney failure patients undergoing maintenance haemodialysis at the Renal and Dialysis unit of Korle-Bu Teaching Hospital (KBTH). Clinical and demographic data were collected and genomic DNA isolated. Samples were genotyped for specific SNPs using Polymerase Chain Reaction-Restriction Fragment Length Polymorphism (PCR-RFLP).

**Results:**

Participants, 58/82 (70.73%) harbored the wildtype CYP3A5*1/*1 AA genotype, 20/82 (24.39%) carried the heterozygous CYP3A5*1/*3 AG genotype, and 4/82 (4.88%) had the homozygous mutant CYP3A5*3/*3 GG genotype. Also, 6/82 (7.32%) carried the wildtype AA genotype, 11/82 (13.41%) had the heterozygous AG genotype, and 65/82 (79.27%) harbored the homozygous mutant GG genotype of CYP3A4*1B (-290 A>G). For *MDR1_Ex21 (2677 G>T)*, 81/82 (98.78%) carried the wildtype GG genotype, while 1/82 (1.22%) had the heterozygous GT genotype. For *MDR1_Ex26 (3435 C>T)*, 63/82 (76.83%) had the wildtype CC genotype, while 18/82 (21.95%) carried the heterozygous CT genotype, and 1/82 (1.22%) harbored the mutant TT genotype.

**Conclusion:**

SNPs in CYP3A4, CYP3A5, and MDR1 genes in a population of Ghanaian kidney failure patients were described. The varying SNPs of the featured genes suggest the need to consider the genetic status of Ghanaians kidney failure patients prior to transplantation and tacrolimus therapy.

## Introduction

The burden of chronic kidney disease (CKD) and kidney failure in Ghana is on the ascendency, with the prevalence of CKD estimated at 13.3% [1, 2]. Patients with CKD who progress to kidney failure require life sustaining kidney replacement therapy (KRT) which is almost exclusively available in Ghana as haemodialysis [3]. Kidney transplantation is however generally considered the best KRT option for patients with irreversible kidney failure due to its relative cost efficiency compared to other KRT methods currently available (haemodialysis and peritoneal dialysis), as well as its superiority in terms of survival and quality of life [4, 5].

Despite the obvious benefits of kidney transplantation, its implementation as the standard of care has been fraught with significant challenges. These are mainly to do with the optimal use of immunosuppressant medications to prevent allograft rejection with minimal adverse effects and the difficulty in acquiring kidneys for transplantation [4]. In Ghana these challenges are further compounded by the lack of a national kidney transplantation programme [3] and the high cost of kidney transplantation in a low middle-income country with the majority of its kidney failure patients being young and constrained financially by their illness in their economically productive years [2, 5]. Kidney transplants have however been done in Ghana, nonetheless. Between the years 2008 and 2014 a total of 17 live donor kidney transplants were successfully performed in Ghana [5, 6] and there is evidence confirming that in 2017 there were 24 patients with functional kidney allografts [3]. These figures, though significantly inadequate, demonstrate a steady incremental improvement in the applicability and utilisation of kidney transplantation in Ghana. It is likely that soon, the requisite legal, logistic, financial, and human resources to run a national kidney transplantation programme will be in full existence in Ghana, and kidney transplantation will become a viable KRT option with a resultant increase in the number of kidney transplant recipients.

Tacrolimus is a key immunosuppressant drug used to induce immune tolerance and prevent allograft rejection in most post kidney transplant immunosuppression protocols used globally [7–9]. It belongs to the Calcineurin Inhibitor family of drugs together with Cyclosporine, and its mechanism of action is to bind to a specific intracellular immunophilin called FK506-binding protein (FKBP12) resulting in the formation of a complex which inhibits calcineurin [7]. Calcineurin is a calcium-calmodulin dependent serine/threonine phosphatase which dephosphorylates and activates the transcription factor Nuclear Factor of Activated T-cells (NFATc). NFATc contributes to the production of cytokines which ultimately result in T cell activation and consequent allograft rejection [10, 11].

Tacrolimus, despite its well documented efficacy with regards to preventing allograft rejection, is a difficult drug to use in clinical practice. This is because of its narrow therapeutic window, which is further compounded by significant inter and intra-patient pharmacokinetic variability [10, 12, 13]. There is thus a chance of kidney transplant recipients being outside the narrow therapeutic window both in the early post-operative period, with a resultant delay in reaching an optimal steady state concentration per dose ratio (C/D ratio, and during the maintenance phase following kidney transplantation [10]. This puts kidney allograft recipients at a continued risk of either under immunosuppression resulting in allograft rejection or the adverse effects of toxic levels of tacrolimus. This risk is generally mitigated in practice by using therapeutic drug monitoring (TDM) [14]. This method results in clinical decisions with regards to Tacrolimus dosing being taken reactively or in retrospect rather than in real time or at the very least prospectively, considering the high stakes involved in terms of allograft rejection or adverse effects from toxicity. Attempts to improve on the current standard of care (TDM) have led to extensive research into the pharmacokinetics and pharmacodynamics of Tacrolimus, in a bid to better predict the effective concentration achieved per dose, establish an optimal steady state concentration more rapidly post kidney transplantation and maintain patients within the therapeutic window to prevent allograft rejection and reduce the incidence of adverse effects. Tacrolimus is mainly metabolised by enzymes belonging to the cytochrome P450 CYP3A group of enzymes, which include CYP3A5 and CYP3A4 notwithstanding other enzymes which belong to the group but are not of significant relevance to the metabolism of Tacrolimus [15, 16]. The P-glycoprotein (ABCB1/MDR1) which functions as an efflux pump has also been found to affect both the absorption and excretion of Tacrolimus metabolites [17, 18]. Single-nucleotide polymorphisms (SNPs) in the genes that code for these enzymes have been demonstrated to affect the concentration of Tacrolimus achieved per dose albeit the application of these findings have yielded mixed results in clinical trials, due to factors other than genetics also playing a significant role in the C/D ratio of Tacrolimus [13, 19–22]. For instance, whereas CYP3A5*1 form of the gene expresses large amounts of CYP3A5, the variant form of the gene, CYP3A5*3, results in the absence of a functional CYP3A5 [23, 24]. Compared to carriers of CYP3A5*1, kidney failure patients harboring the CYP3A5*3 form were reported to be associated with increased risk of early renal glomerular injury upon receiving a kidney transplant [23, 24]. Also, variations in the MDR1 gene C3435T and G2677T have been reported as risk factors for acute rejection in kidney transplant recipients [25]. So, for a better outcome, tacrolimus treatment may be adapted to the recipient genotype [25].

How will alternate forms of tacrolimus metabolizing enzymes affect tacrolimus dose requirements and impact treatment outcome of potential Ghanaian kidney transplant recipients?

This study described the allele and genotype frequencies of SNPs in CYP3A5, CYP3A4, and MDR1 genes that affect the pharmacokinetics of Tacrolimus in a population of Ghanaian patients with kidney failure. Although their application to clinical practice is still being perfected; this study provides valuable knowledge that may improve the management of kidney transplant patients in Ghana through genomic medicine.

## Materials and methods

### Study design

This was a hospital based cross-sectional study which was conducted at the Renal and Dialysis unit of the Korle Bu Teaching Hospital (KBTH) located in Accra, Ghana. Patients who met the inclusion criteria and provided their written informed consent were recruited into the study using a consecutive sampling method. The period of recruitment was from 2016 to 2017. The study was approved by the Ethics and Protocol Review Committee of the University of Ghana, College of Health Sciences: reference number CHS-Et/M.1-P2.8/2016–2017.

### Patients

A total of eighty-two (82) kidney failure patients undergoing maintenance haemodialysis at the Renal and Dialysis unit KBTH were recruited into the study over the period. To meet the study inclusion criteria, patients were required to be above the age of 18 years, have kidney failure and be on maintenance haemodialysis at the study site. It was also a requirement for them to be of Ghanaian nationality. Maintenance haemodialysis was considered as having been on a minimum dialysis frequency of two times a week continuously over the three-month period prior to recruitment.

### DNA extraction

Genomic DNA was extracted from whole blood samples using a commercial extraction kit; Zymo Research Quick-gDNA MiniPrep kits (Inqaba Biotec Ltd, SA) following the manufacturer’s protocol. The eluted gDNA was stored at -20^O^C and used to genotype specific alleles.

### Genotyping

Genotyping of the specific alleles CYP3A5*3 (6986 A>G), CYP3A4*1B (-290 A>G), *MDR1_Ex12 (1236 C>T)*,* MDR1_Ex26 (3435 C>T)*,* MDR1_Ex21 (2677 G>A)*,* MDR1_Ex21 (2677 G>T)* were performed by Polymerase Chain Reaction-Restriction Fragment Length Polymorphism (PCR- RFLP) as previously described by Sarasamma et al. [26] with some modifications. Briefly, in each PCR reaction, 5 µl of genomic DNA, 2 µl each of 10µM forward and reverse primers designed to amplify a 293 bp fragment, and 10 µl of One Taq Quick-Load 2X Commercial Master Mix, and 1 µl of nuclease-free water were used in a final reaction volume of 20 µl. PCR was performed in a Thermos Scientific thermal cycle (cycling conditions were initial denaturation at 94^o^C for 1 min, annealing at 58 ^o^C for 1 min (60^o^C for 1 min for MDR1), extension at 72 ^o^C for 1 min, and a final extension step at 72 ^o^C for 7 min; the number of cycles was 35. The PCR product was analyzed on a 2% agarose EDTA gel with ethidium bromide staining and bands were analyzed by gel documentation system. Aliquots of the PCR products were digested with appropriate restriction enzymes Ssp I, Pst I, Hae III, Bsr I, Ban I, and Mbo I (New England Biolabs), [for CYP3A5*3 (6986 A>G), CYP3A4*1B (-290 A>G), MDR1_Ex12 (1236 C>T), MDR1_Ex21 (2677 G>A), MDR1_Ex21 (2677 G>T), and MDR1_Ex26 (3435 C>T) respectively]. A 10 µl aliquot of PCR product, 1 µl endonuclease, 2 µl 10X buffer R, and 7 µl nuclease-free water, was incubated for 3 h at 37ºC and analyzed on 3% agarose gel with ethidium bromide staining and bands detected by UV trans-illuminator.

### Statistical analyses

Data obtained from both administered questionnaire and laboratory genotyping were analyzed using Stata 14.0^®^ and summarized as frequencies and proportions. Chi-squared test (p2 + 2pq + q2 = 1) was used to determine consistency with Hardy-Weinberg equilibrium.

## Results

### Characteristics of study participants

Of the eighty-two patients recruited, 71.26% (*n* = 62) were males and 28.74% (*n* = 25) were females. The mean age was 46±14.39 years. Majority of the patients (43.68%) were aged between 41 and 60 years.

### Genotypic profiles

A total of 6 SNPs in 5 candidate genes were studied. CYP3A4*1B (-290 A > G) had the highest variant G allele frequency of 85.98% and genotypic frequency for a homozygous mutant gene (79.27%). *MDR1_Ex12 (1236 C > T) and MDR1_Ex21 (2677 G > A)* had the lowest allelic frequency (0%) and the highest genotypic frequency for the wild type (100%). The detailed profiles of the SNPs studied are presented in Table 1.

**Table 1:** Allele and genotype frequencies of CYP3A5*3 (6986 A>G), CYP3A4*1B (-290 A>G), MDR1_Ex12 (1236 C>T), MDR1_Ex21 (2677 G>A), MDR1_Ex21 (2677 G>T) and MDR1_Ex26 (3435 C>T) genes

			Kidney failure patients
**Gene**			*N* = 82
			n, %
CYP3A5*3 (6986 A>G)	Allele	A	136 (82.93)
		G	28 (17.07)
	Genotype	AA	58 (70.73)
		AG	20 (24.39)
		GG	4 (4.88)
CYP3A4*1B (-290 A>G)	Allele	A	23 (14.02)
		G	141 (85.98)
	Genotype	AA	6 (7.32)
		AG	11 (13.41)
		GG	65 (79.27)
*MDR1_Ex12 (1236 C>T)*	Allele	C	164 (100)
		T	0 (0)
	Genotype	CC	82 (100)
		CT	0 (0)
		TT	0 (0)
*MDR1_Ex21 (2677 G>A)*	Allele	G	164 (100)
		A	0 (0)
	Genotype	GG	82 (100)
		GA	0 (0)
		AA	0 (0)
*MDR1_Ex21 (2677 G>T)*	Allele	G	163 (99.39)
		T	1 (0.61)
	Genotype	GG	81 (98.78)
		GT	1 (1.22)
		TT	0 (0)
*MDR1_Ex26 (3435 C>T)*	Allele	C	144 (87.80)
		T	20 (12.20)
	Genotype	CC	63 (76.83)
		CT	18 (21.95)
		TT	1 (1.22)

CYP3A5*3(6986 A>G) and CYP3A4*1B (-290 A>G) (A = Wild-type allele,

G = Variant allele, AA = Homozygous-wildtype, AG = Heterozygous

GG = Homozygous mutant); MDR1_Ex12 (1236 C>T) and MDR1_Ex26

(3435 C>T) (C = Wildtype allele, T = Variant allele, CC = Homozygous wildtype,

CT = Heterozygous, TT = Homozygous mutant); MDR1_Ex21 (2677 G>A)

(G = Wild-type allele, A = Variant allele, GG = Homozygous-wildtype, GA=Heterozygous, AA = Homozygous mutant); MDR1_Ex21 (2677 G>T) (G=Wildtype allele, T = Variant allele, GG = Homozygous-wildtype, GT=Heterozygous, TT = Homozygous mutant)

#### CYP3A5*3 (6986 A>G)

Most participants, 136/164 (82.93%) had the wildtype A allele, a few, 28/164 (17.07%) carried the variant G allele, 58/82 (70.73%) harbored the wildtype CYP3A5*1/*1 AA genotype, 20/82 (24.39%) carried the heterozygous CYP3A5*1/*3 AG genotype, and 4/82 (4.88%) had the homozygous mutant CYP3A5*3/*3 GG genotype.

### CYP3A4*1B (-290 A>G)

Most study participants, 141/164 (85.98%) had the variant G allele frequency while a few participants, 6/82 (7.32%) carried the wildtype AA genotype, 11/82 (13.41%) had the heterozygous AG genotype, and 65/82 (79.27%) harbored the homozygous mutant GG genotype of CYP3A4*1B (-290 A>G).

### MDR1 haplotypes

All study participants, 82/82 (100%) had the wildtype *MDR1_Ex12 (1236 C>T)* allele C, and CC genotype, and wildtype *MDR1_Ex21 (2677 G>A)* allele G, and GG genotype, (Table 1). For *MDR1_Ex21 (2677 G>T)*, 163/164 (99.39%) had the wildtype G allele, 1/164 (0.61%) harbored the variant T allele, 81/82 (98.78%) carried the wildtype GG genotype, while 1/82 (1.22%) had the heterozygous GT genotype. For *MDR1_Ex26 (3435 C>T)*, 144/164 (87.80%) harbored the wildtype C allele, 20/164 (12.20%) carried the variant T allele, 63/82 (76.83%) had the wildtype CC genotype, while 18/82 (21.95%) carried the heterozygous CT genotype, and 1/82 (1.22%) harbored the mutant TT genotype.

## Discussion

A total of 82 kidney failure patients on maintenance haemodialysis were recruited into this hospital-based cross-sectional study. The mean age was 46±14.39 years and majority of the patients (43.68%) were aged between 41 and 60 years. This reflects the relatively young age distribution of Ghanaian patients with kidney failure compared to other populations [27]. The benefit of kidney transplantation in this young group of patients cannot be overemphasised and it is important that their post-transplant care is optimal to ensure good outcomes.

CYP3A5 plays a major role in the metabolism of Tacrolimus. Polymorphisms of the CYP3A5 gene have been studied extensively, and account for 40–50% of the variability observed in the dose of tacrolimus required to achieve a target effective concentration [28, 29]. CYP3A5*3, CYP3A5*6 and CYP3A5*7 are examples of SNPs that have been studied, with CYP3A5*3 being the most widely researched. CYP3A5*3 is an A to G mutation at position 6986 [30] which results in loss of function of the resultant CYP3A5 protein. This loss of function is most pronounced in the homozygous mutant, followed by the heterozygous mutant and is absent in the homozygous wild-type; with the effect being a lower tacrolimus dose requirement to achieve the target effective concentration [31].

The present study observed the variant allele frequency CYP3A5*3 as 17.07% and this is similar to the 15% reported in a previously published data from a Ghanaian population [32]. CYP3A5*3 (6986 A > G) was reported in previous studies as ranging from 4 to 81% in Africans and this is congruent with what was observed in this study. Compared to East Africa, Sub-Saharan Africa has the lowest CYP3A5*3 frequencies [33]. In Africans and African Americans, the agreement between the presence of the *3 allele and increased CYP3A5 expression is less robust compared to Caucasians. This may be due to additional polymorphisms in Africans (e.g., *6 and *7 which occur at frequencies of 5–25% and 0–21%, respectively) as variants *6 and *7 have both been found in individuals homozygous for the *3 allele [34].

Most participants in the present study had the homozygous wildtype genotype of CYP3A5, with others harboring the heterozygous genotype, and a few carrying the homozygous mutant. This finding was similar to a previous study which reported CYP3A5 homozygous wildtype (*1/*1), heterozygous (*1/*3), and homozygous mutant (*3/*3) genotypes at 70.5%, 29%, and 0.5% respectively in a Ghanaian population [32]. Sarasamma et al. observed that transplant rejection cases were notably higher in carriers of CYP3A5*1/*1 compared with those who harbored CYP3A5*1/*3, and CYP3A5*3/*3 genotypes respectively in South Indian kidney transplant recipients [26]. Furthermore, they reported significantly higher tacrolimus dosage ratio in CYP3A5*1/*1 compared with CYP3A5*3/*3, though lower doses were reported in CYP3A5*1/*3 carriers [26]. Muller et al. also reported a two-fold increase in tacrolimus dose requirement for carriers of CYP3A5*1/*1 and CYP3A5*1/*3 compared with those who harbor CYP3A5*3/*3 in South African kidney transplant recipients [35]. Individuals carrying the mutant CYP3A5 enzymes are at risk of experiencing the adverse effects associated with drug overdose.

The variant allele frequency of CYP3A4*1B observed in this study was 85.98% and this compares with 69% and 81% earlier reported by two independent studies in Ghanaians [36, 37]. These findings are consistent with the allele frequency range of 66–86% previously observed in other African populations [34]. Previous studies reported an observed variant allele frequency of 72%, 87%, and 78%, for CYP3A4*1B in Guinea-Bissau [38], Nigeria [39], and Senegal [37] respectively. However, this allele was not detected in Africans from North Sahara [40].

This SNP results in a gain-of-function mutation that increases the activity of the CYP3A4 enzyme [41]. CYP3A4*1B has been found to be in linkage disequilibrium with CYP3A5*3 and this could be what accounts for its gain of function effect instead of the independent effect of the SNP [42, 43]. Most participants in this study carried the homozygous mutant genotype of CYP3A4, while others harbored the heterozygous genotype, and a few had the wildtype genotype. This finding was similar to a previous study which reported CYP3A4*1B homozygous mutant (*1B/*1B) genotype, heterozygous (*1/*1B) genotype, and homozygous wildtype (*1/*1) genotype, at 50%, 42%, 7% respectively in a Ghanaian population [32]. A meta-analysis by Shi et al. suggest that the CYP3A4*1B genetic polymorphism influences the weight-adjusted tacrolimus daily dose and the tacrolimus trough concentration (C0/Dose ratio) in adult kidney transplant recipients [44]. They reported a significantly higher tacrolimus trough concentration/weight-adjusted tacrolimus daily dose ratio (C0/Dose ratio) in CYP3A4*1/*1 recipients than in CYP3A4*1B carriers [44]. Mutation in the CYP3A4 gene is linked to enhanced activity/up-regulation of the enzyme, causing rapid metabolism of medications including tacrolimus [44]. The reported SNPs of CYP3A4 in kidney failure patients in the present study may be important in tacrolimus dose requirement in transplant recipients.

The variant allele frequencies of the MDR1 SNPs were relatively low in this study with MDR1_Ex26 (3435 C > T) having the highest frequency at 12.20%. Most participants had 100% wildtype *MDR1_Ex12 (1236 C>T)* and *MDR1_Ex21 (2677 G>A)* genotypes, 98.9% wildtype *MDR1_Ex21 (2677 G>T)* genotype, and 74.71%, 24.14%, and 1.15% for *MDR1_Ex26 (3435 C>T)* wildtype, heterozygous, and homozygous genotypes respectively. The effect of MDR1 SNPs on tacrolimus metabolism has proven to be uncertain and several studies have not shown an association between MDR1_Ex26 (3435 C > T) and Tacrolimus pharmacokinetics [28, 43, 45–47]. However, the C allele, homozygous CC, and heterozygous CT genotypes of MDR1 C3435T and the T allele, heterozygous GT, and homozygous TT genotypes of MDR1 G2677T gene polymorphism were reported by Korkor et al. as likely risk factors for acute rejection due to their effect on tacrolimus pharmacokinetics [48]. Kravljaca et al. reported that carriers of 2677 G > T/A and C3435T homozygous TT required a higher tacrolimus dose than those with the wildtype or heterozygous genotypes [49], and that this may help in the prevention of tacrolimus nephrotoxicity early after transplantation.

Tailoring tacrolimus therapy based on the recipient genotype may be required for better outcome.

The data from our study paves the way for further research in Ghanaian kidney transplant patients to determine the optimal dose of Tacrolimus to prevent allograft rejection in a personalised and pre-emptive manner. It has however been realised that gene-based Tacrolimus prescribing per se does not result in more effective Tacrolimus dosing [20, 31, 50]. Computer-assisted dose individualisation software that incorporate other variables like age, body surface area, plasma albumin level and ethnicity [51] have shown significant promise in practice in terms of applying pharmacogenetics to improve on the current standard TDM [52]. This study provides the seminal evidence that will form the basis of investigating the applicability of algorithm-based Tacrolimus dosing in Ghanaian kidney failure patients.

### Limitation

Most kidney failure patients were reluctant to join the study due to lack of feedback from previous studies they participated in.

## Conclusion

Most of the kidney failure patients in the present study harbored SNPs of CYP3A4, CYP3A5, MDR1 C3435T, and MDR1 G2677T, that could affect tacrolimus dose requirement in transplant recipients. The varying polymorphisms of the featured genes suggest the need to consider the genetic status of Ghanaians kidney failure patients prior to transplantation and tacrolimus therapy.

## Data Availability

The datasets used and/or analyzed during the current study are available from the corresponding author on reasonable request.

## Abbreviations

CYP3A4: Cytochrome P450 3A4 gene
CYP3A5: Cytochrome P450 3A5 gene
MDR1: Multi-Drug Resistance 1 gene
SNPs: Single Nucleotides Polymorphisms
PCR-RFLP: Polymerase Chain Reaction-Restriction Fragment Length Polymorphism
CKD: Chronic Kidney Disease
DNA: Deoxyribonucleic acid
gDNA: Genomic Deoxyribonucleic acid
EDTA: Ethylenediaminetetraacetic acid
Ssp I: A restriction enzyme purified from Sphaerotilus species
Pst I: A type II restriction endonuclease isolated from the Gram-negative species, Providencia stuartii
Hae III: A restriction enzyme purified from Haemophilus aegypticus
Bsr I: A restriction enzyme purified from Bacillus stearothermophilus (C. Polisson)
Ban I: A restriction enzyme purified from Bacillus aneurinolyticus
Mbo I: A restriction enzyme purified from Moraxella bovis
